# Influence of the proximal screws of buttress plates on the stability of vertical femoral neck fractures: a finite element analysis

**DOI:** 10.1186/s12891-020-03853-7

**Published:** 2020-12-12

**Authors:** Shi Zhan, Dajun Jiang, Jian Xu, Ming Ling, Kai Yang, Yuehua Li, Weitao Jia, Hai Hu, Changqing Zhang

**Affiliations:** 1 Orthopedic Biomechanical Laboratory of Department of Orthopedic Surgery, Shanghai Jiao Tong University Affiliated Sixth People’s Hospital, NO. 600, Yishan Rd., Shanghai, 200233 People’s Republic of China; 2 Department of Orthopedic, Fudan University Affiliated Huadong Hospital, Shanghai, 200040 People’s Republic of China; 3 Radiology Department, Shanghai Jiaotong University Affiliated Sixth People’s Hospital, Shanghai, 200233 People’s Republic of China; 4 Department of Orthopedic Surgery, Shanghai Jiaotong University Affiliated Sixth People’s Hospital, Shanghai, 200233 People’s Republic of China

**Keywords:** Vertical femoral neck fracture, Buttress plate, Proximal screw, Biomechanics, Finite element analysis

## Abstract

**Background:**

The treatment of vertical femoral neck fractures (vFNFs) in young patients remains challenging, with a high complication rate by using traditional techniques. The use of cannulated screws (CSs) combined with a buttress plate represents an alternative approach for treating vFNFs. However, the biomechanical influence of the use or non-use of the proximal screws of buttress plates on vFNFs stability remains unclear. This study aims to analyse the biomechanics of buttress plate fixation with or without the use of proximal screws through finite element analysis (FEA) to further understand this approach.

**Methods:**

We built five vFNFs (Pauwels angle 70°) finite element models treated using three cannulated screws (CS group) or three cannulated screws plus a locking buttress plate (buttress group). In the buttress group, use or non-use of proximal screws was carried out on two types of plates (4-hole & 6-hole). The following seven parameters were analysed to compare biomechanical properties of the five models: the stiffness; the maximal stress of the plate system (plate and screws), CSs and bone (MPS, MCS, MBS); the maximal displacement of internal fixations (plate system & CSs) and bone (MIFD, MBD); and the maximal relative displacement of interfragments (MID).

**Results:**

Compared with CS model, the buttress models exhibited improved biomechanical properties, with increased stiffness and decreased MCS, MBS, MIFD, MBD and MID. The models fixed using buttress plates combined with a proximal screw showed greater stiffness (+ 3.75% & + 8.31% vs + 0.98% & + 4.57%) and MPS (795.6 & 947.2 MPa vs 294.9 & 556.2 MPa) values, and smaller MCS, MBS, MIFD, MBD and MID (− 3.41% to − 15.35% vs − 0.07% to − 4.32%) values than those using the same length plates without a proximal screw.

**Conclusions:**

Based on the FEA results, buttress plates can improve construct mechanics, help to resist shear force and prevent varus collapse; under the modelling conditions, the use of a proximal screw on buttress plate may be a key technical feature in improving anti-shearing ability; additionally, this screw may be essential to reduce stress and prevent re-displacement of cannulated screws and fracture fragments.

## Highlights

We describe biomechanical analysis of the buttress plate.

The buttress plate provides improved initial stability of vertical femoral neck fractures.

The proximal screw of the buttress plate is key for mechanical transmission of force when treating vertical femoral neck fractures.

## Introduction

The treatment of vertical femoral neck fractures (vFNFs, Pauwels type III) in young adults is challenging because this type of fracture, which usually results from high-energy trauma, is subject to high shear forces, leading high rates of non-union and osteonecrosis [1, 2]. Additionally, most vFNFs are comminuted and are mostly centred in the inferior and posterior quadrants, resulting in difficulties reconstituting the bony buttress [3]. To achieve satisfactory treatment of this type of fracture, anatomic reduction and stable internal fixation are necessary [4]. However, failure after fixation has been seen using essentially all devices, including cannulated screws in various configurations, sliding hip screws with or without additional derotation screws, cephalomedullary nails, and proximal femoral locking plates, and the failure rate of surgery for vFNFs remains high [1, 3, 5].

The potential application of a buttress plate to improve the fixation stability of vFNFs was first suggested by Mir and Collinge [6]. With the help of the buttress plate, the shear force can be converted into compressive forces to achieve medial buttress stability. The utility and safety of the buttress plate have been validated in previous studies [7–10]. However, there is no consensus on the use of a proximal screw on the buttress plate, there are no detailed usage guidelines. On the one hand, Mir and Collinge hypothesized the non-use of proximal screws because the use of a buttress plate may be sufficient to reduce shear effects [6]. Additionally, to reproduce a specific clinical situation, Giordano et al. did not use a proximal screw in the head [11]. On the other hand, Kunapuli et al. performed an experimental study by using cannulated screws or a DHS, augmented by a 2.7 mm locking plate with a proximal screw, resulting in positively [7]. Furthermore, Ye et al. [9] also used a proximal screw and indicated that the screw proximal to the fracture line should be directed cephalad into the femoral head to avoid crossing the fracture line, only changing the locking plate to an unlocking plate. They argued that locked screws may prevent the dynamic compression between the fracture fragments postoperatively and may increase the risk of non-union; however, there were still 3 out of 27 patients who experienced implant failure, and associated with femoral neck shortening. Thus, how the use of a proximal screw on the buttress plate affects the overall stability of surgically treated vFNFs is an interesting question. The present study aimed to evaluate the benefits of the buttress plate and its implications in vFNFs treatment and to verify the influences of the use of a proximal screw on the stability of vFNFs through finite element analysis. This work will help orthopaedic surgeons to further understand the biomechanical properties of buttress plates and proximal screws, enabling appropriate clinical decision making for the treatment of vFNFs.

## Materials and methods

### Modelling of femoral neck fractures

Computed tomography images (SOMATOM Definition AS1; Siemens, thickness, 0.6 mm; resolution, 512 × 512 pixels) of a Sawbone femur (Model 3402, 4th Generation Sawbone, Vashon, WA, USA) were obtained and imported into Mimics 19.0 (The Materialise Group, Leuven, Belgium) to create a three-dimensional model. The model was cut with a modified Pauwels angle of 70 degrees in 3-Matic (version 11.0 Materialise, Leuven, Belgium) to simulate a Pauwels type III femoral neck fracture. Sawbone femurs were chosen because they have been validated to minimize individual variations and have been confirmed to be a suitable replacement for cadavers [12–14].

### Modelling of internal fixation for femoral neck fractures

**Fig. 1:**
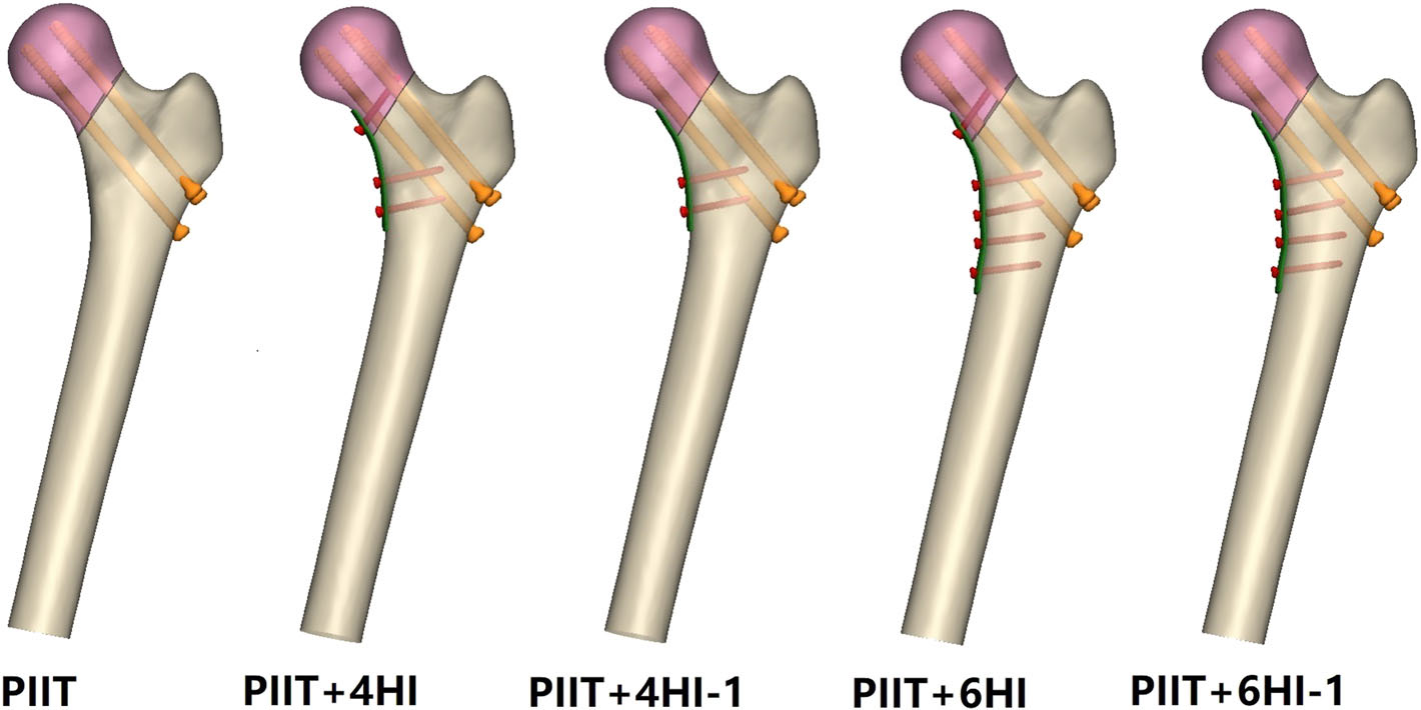
Diagrams of the five models. PIIT represents femoral neck fracture fixed using three cannulated screws in a parallel, inverted, isosceles triangular configuration; PIIT+ 4HI represents PIIT combined with a four-hole buttress plate and proximal screw; PIIT+ 4HI-1 represents PIIT combined with a four-hole buttress plate without a proximal screw; PIIT+6HI and PIIT+6HI-1 represents PIIT combined with a six-hole buttress plate with and without a proximal screw, respectively

Since four to six-hole buttress plates are commonly used in the clinic [9] and thin plates may reduce the possible irritation to medial femoral neck structures [7], four- and six-hole 2.7 mm locking plates [7, 10] were adopted in this study. The buttress plate, 3.5 mm screws (plate screw) and 6.5 mm cannulated screws (Stryker, Mahwah, NJ, USA) were created in SolidWorks2014 (DS SolidWorks Corp, Waltham, MA, USA) based on real geometrical dimensions. The three cannulated screws, of which two 90 mm cannulated screws were inserted into the femoral proximal region and one 100 mm cannulated screw was inserted into the distal region, were inserted in parallel to form an inverted isosceles triangular configuration (PIIT). And all the buttress plates were placed in a standard medial position, as shown in previous studies [9, 10]. Five internal fixation models were built (Fig. 1) and named PIIT, PIIT+ 4HI, PIIT+4HI-1, PIIT+6HI and PIIT+6HI-1. We considered PIIT as the control because it is commonly used in clinical practice and is considered to provide good biomechanical stability [15]. In PIIT+ 4HI, PIIT+4HI-1, PIIT+6HI and PIIT+6HI-1, a four- or six-hole buttress plate was applied over the apex of fracture with or without a screw in the proximal region (Fig. 1).

### Material properties and boundary conditions

The Sawbone femur was assumed to be linear, elastic, and isotropic. For cortical and cancellous bones, the Young’s moduli (E) were 17.0 and 0.0155 GPa and Poisson’s ratios (v) were 0.3 and 0.3, respectively. The number of nodes (ranging from 135,967 to 156,165) and elements (ranging from 601,304 to 672,502) for all models were recorded and are detailed in Table 1. A four-node tetrahedron body element (C3D4) was used for the bone, plates, and screws, similar to other studies [16, 17].

**Table 1:** The counts of element and node of five models

Model	PIIT	PIIT+4HI	PIIT +4HI-1	PIIT +6HI	PIIT +6HI-1
Element	601,304	646,021	637,648	672,502	664,117
Node	135,967	148,678	146,294	156,165	153,778

Abbreviations: *PIIT* Parallel, inverted, isosceles triangular plate configuration; *PIIT+4HI* Parallel, inverted, isosceles triangular plate configuration combined with a four-hole buttress plate and proximal screw; *PIIT+4HI-1* Parallel, inverted, isosceles triangular plate configuration combined with a four-hole buttress plate without a proximal screw; *PIIT+6HI and PIIT+6HI-1* Parallel, inverted, isosceles triangular plate configuration combined with a six-hole buttress plate with and without a proximal screw

**Fig. 2:**
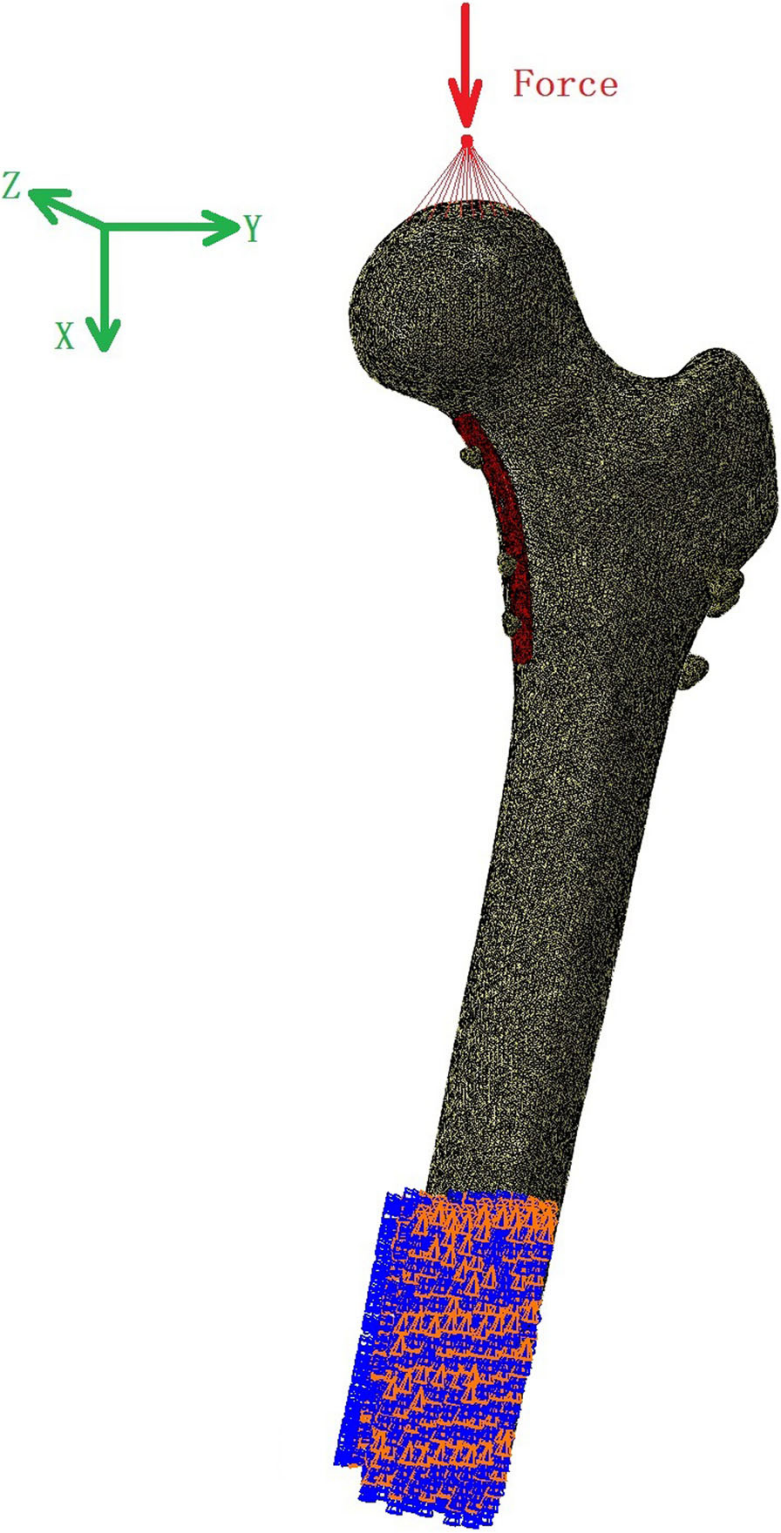
The loading diagram. A 2100 N load corresponding to 300% body weight was uniformly applied to the weight-bearing region of the femoral head along the mechanical axis. Freedom of the distal femur, which had a length of 108 mm, was bounded

The slipping friction factor of the interface between two fracture surfaces was set to 0.46, and the corresponding factor for the interface of the bone and plate was set to 0.3 [18]. A 2100 N load corresponding to 300% body weight [10, 19] was uniformly applied to the weight-bearing region of the femoral head along the mechanical axis (Fig. 2). Freedom of the distal femur, which had a length of 108 mm, was bounded. All models were simulated using Abaqus 6.13 (Simulia Corp, Providence, RI, USA).

### Parameters for analysis

The following parameters were used for analysis: the stiffness (representing the whole stability [20, 21]), the maximal stress of the plate system (plate and screws), cannulated screws (CS), and bone (MPS, MCS, and MBS, respectively); and the maximal displacement of internal fixation (plate system and CS) and bone (MIFD and MBD, respectively), which were the same as in the previous study [10]. The maximal relative displacement of interfragments (MID) (representing the interfragmentary shear stability [22]) was also analysed. All values were compared between the buttress group and the CS group (Table 2) except for MPS, and the corresponding variation rates were calculated by the following formula:

$$ \mathrm{VR}=\frac{\mathrm{BV}-\mathrm{CV}}{\mathrm{CV}}\times 100\% $$

where VR = variation rate, BV = value for the buttress group, and CV = value for the CS group.

**Table 2:** Categorizing of five models and their simulated results of seven parameters

	CS	Buttress			
Parameters	PIIT	PIIT+4HI	PIIT+4HI-1	PIIT+6HI	PIIT+6HI-1
Stiffness (N/mm)	1511.0	1567.6 (+ 3.75%)	1525.8 (+ 0.98%)	1636.5 (+ 8.31%)	1580.1 (+4.57%)
MPS (MPa)	/	795.6	294.9	947.2	556.2
MCS (MPa)	336.3	313.2 (−6.87%)	335.9 (−0.12%)	304.3 (−9.52%)	335.1 (−0.36%)
MBS (MPa)	138.8	118.4 (−14.70%)	138.7 (−0.07%)	117.5 (−15.35%)	138.4 (−0.29%)
MIFD (mm)	1.262	1.219 (−3.41%)	1.251 (−0.87%)	1.169 (−7.37%)	1.209 (−4.2%)
MBD (mm)	1.342	1.294 (−3.58%)	1.329 (−0.97%)	1.240 (−7.60%)	1.284 (− 4.32%)
MID (mm)	7.784E−2	6.742E−2 (−13.39%)	7.777E− 2 (−0.09%)	6.626E−2 (−14.88%)	7.742E−2 (−0.54%)

Abbreviations: *CS* Cannulated screw; *MPS* Maximal stress of plate and screws; *MCS* Maximal stress of cannulated screws; *MBS* Maximal stress of bone; *MIFD* Maximal displacement of internal fixation; *MBD* Maximal displacement of bone; *MID* Maximal relative displacement of interfragments. From the third row on, the values in brackets are the variation rates which were defined in the method

## Results

### Stiffness

The stiffness of the models with a proximal screw was superior to that of models without a proximal screw (Table 2). The variation rates of stiffness in the models with a proximal screw (PIIT+4HI & PIIT+6HI) were + 3.75% and + 8.31%, while those in the models without a proximal screw (PIIT+4HI-1 & PIIT+6HI-1) were + 0.98% and 4.57%. Maximal stiffness was observed in the PIIT+6HI model, with a value of 1636.5 N/mm and a variation rate of + 8.31%. In contrast, PIIT+4HI-1 exhibited the poorest stiffness, with a value of 1525.8 N/mm and a variation rate of + 0.98%.

### Maximal stress

**Fig. 3:**
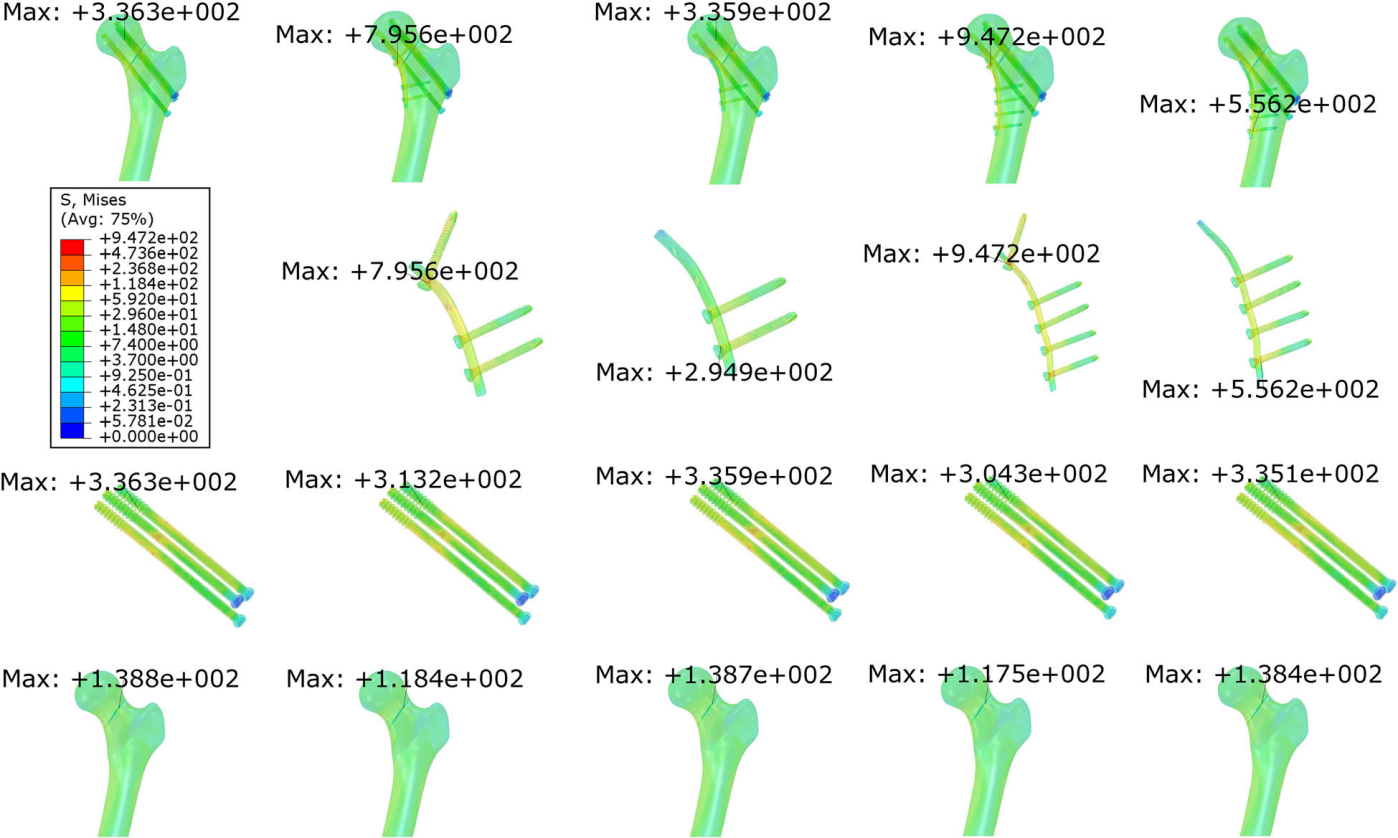
Stress diagrams of the five models. The stresses on the buttress plate and screws on it, cannulated screws, and femora are shown

The maximal stress of the plate system (MPS) in the models with a proximal screw in the buttress group was larger than that in the models without a proximal screw in the same group (795.6 & 947.2 vs 294.9 & 556.2). The maximal stress of cannulated screws (MCS) and the maximal stress of bone (MBS) in the CS group were found to be 336.3 and 138.8 MPa. The maximal and minimal MPS values were 947.2 and 294.9 MPa in PIIT+6HI and PIIT+4HI-1, respectively. Maximal MCS and MBS values occurred in PIIT+4HI-1, with values of 335.9 MPa (variation rate = − 0.12%) and 138.7 MPa (variation rate = − 0.07%), respectively, while minimal MCS and MBS values were observed in PIIT+6HI, with values of 304.3 MPa (variation rate = − 9.52%) and 117.5 MPa (variation rate = − 15.35%). The locations of maximal stress are illustrated in Fig. 3. The MCS and MBS values of the models in the buttress group were all reduced compared to those in the CS group, with variation rates of − 0.07% to − 15.35% (Table 2).

### Maximal displacement

**Fig. 4:**
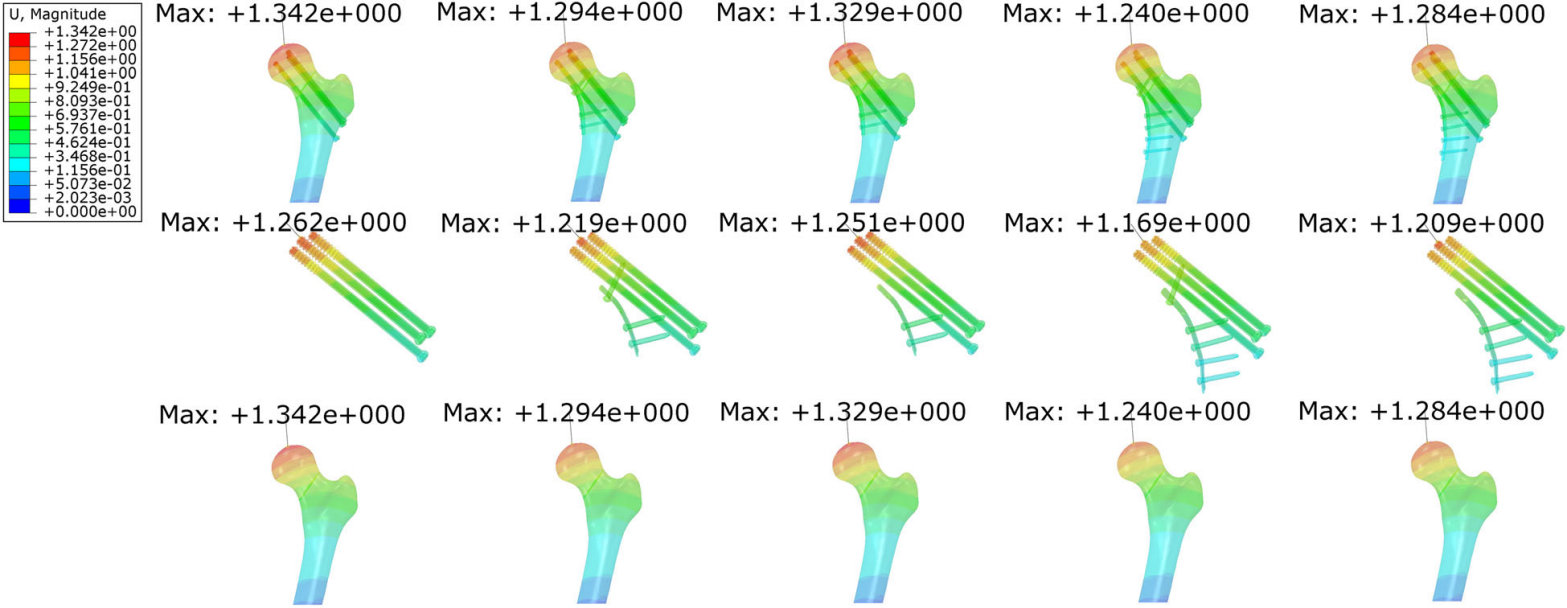
Displacement diagrams of the five models. The displacements on the internal fixations (plate system and cannulated screws) and femora are shown

The maximal displacement of the internal fixation (MIFD) and the maximal displacement of bone (MBD) in the models with a proximal screw in the buttress group were smaller than those in the models without a screw in the same group (Fig. 4 & Table 2). The variation rates of MIFD in the models with a proximal screw (PIIT+4HI & PIIT+6HI) were − 3.41% and − 7.37%, while those in the models without a proximal screw (PIIT+4HI-1 & PIIT+6HI-1) were − 0.87% and − 4.2%. The compared results of MBD regarding the variation rates between models with and without a proximal screw were similar to those of MIFD, with compared values of − 3.58% & -7.6% and − 0.97% & -4.32%. The MID values of the buttress group were 6.742E− 2 mm, 6.626E− 2 mm, 7.777E− 2 mm, and 7.742E− 2 mm for PIIT+4HI, PIIT+6HI, PIIT+4HI-1, and PIIT+6HI-1, respectively. The MID in the CS group was 7.784E− 2 mm. All MID values in the buttress group were lower than those in the CS group, with variation rates ranging from − 0.09% (PIIT+4HI-1) to − 14.88% (PIIT+6HI). The models with a proximal screw (PIIT+4HI & PIIT+6HI) showed smaller MID values than those without (PIIT+4HI-1 & PIIT+6HI-1), of which the variation rates ranged from − 13.39% & -14.88% to − 0.09% & -0.54%, respectively.

## Discussion

The combination of using a buttress plate with three parallel, inverted, and isosceles triangular (PIIT) screws offers an alternative approach for stabilizing fractures with increased resistance to shear forces, with early satisfactory results [6, 7, 9]. For displaced vFNFs, especially those with comminution, this construct can prevent the inferior translation along the fracture line. Open approaches also provide the best opportunity for achieving anatomical reduction, and reduction of the apical fracture spike may dramatically increase the stability [3]. Moreover, capsulotomy allows decompression of the intra-capsular hematoma [6]. As Fig. 3 & Fig. 4 show, in comparison with fixation using three parallel cannulated screws, the buttress plate augmented the stiffness of the structure and reduced the maximal displacement of the internal fixation and the femur, the maximal relative displacement of the fracture fragments (MIFD, MBD, MID) and the shear stress on cannulated screws (MCS) and surrounding bone (MBS). Consequently, the buttress plate improves construct mechanics, helps to resist shear force and prevents varus collapse.

However, historical failure rates still exist [9]. We found that the detailed surgical technique of the buttress plate is obscured without biomechanical evidence, for instance, whether the proximal screw is used [6, 7, 9] and whether the proximal screw is unlocked [9] or locked [7]. In the present study, we aimed to address this uncertainty by comparing four fixed vFNF models that used two types of buttress plates over the apex of the fractures with or without proximal screws on the plates. Our pilot study found that a proximal screw locked to the buttress plate was a key technical feature contributing to transmitting force from the head to the shaft of femur and could reduce stress and displacements on the cannulated screws and bone, as well as reduce the relative displacement of fracture fragments. However, more complicated questions in terms of the “unlocked and locked” problem were associated with static and dynamic fixation concepts and could hardly be answered biomechanically, which only reflects initial stability rather than the healing process. Therefore, this problem should be left out of further studies, and “whether the proximal screw is used” should be the focus, following the fixation model of Kunapuli SC’s study [7].

The stiffness of PIIT (1511.0 N/mm) was found to be similar to that reported in previous biomechanical tests (1418 ± 88 N/mm [20] and 1469.0 ± 113.5 N/mm [21]), indicating that our modelling method is appropriate for the evaluation of vFNFs stability. In terms of the construction choice of CSs, we used PIIT rather than a specific construction in Giordano et al.’s study [11], as PIIT is considered to provide good biomechanical stability and is more commonly used in clinical practice [9, 15]. As our aim was to investigate the biomechanics of the proximal screw, all the buttress plates in our models were placed in a standard medial position [7, 9, 10] different from the previous study [11].

**Fig. 5:**
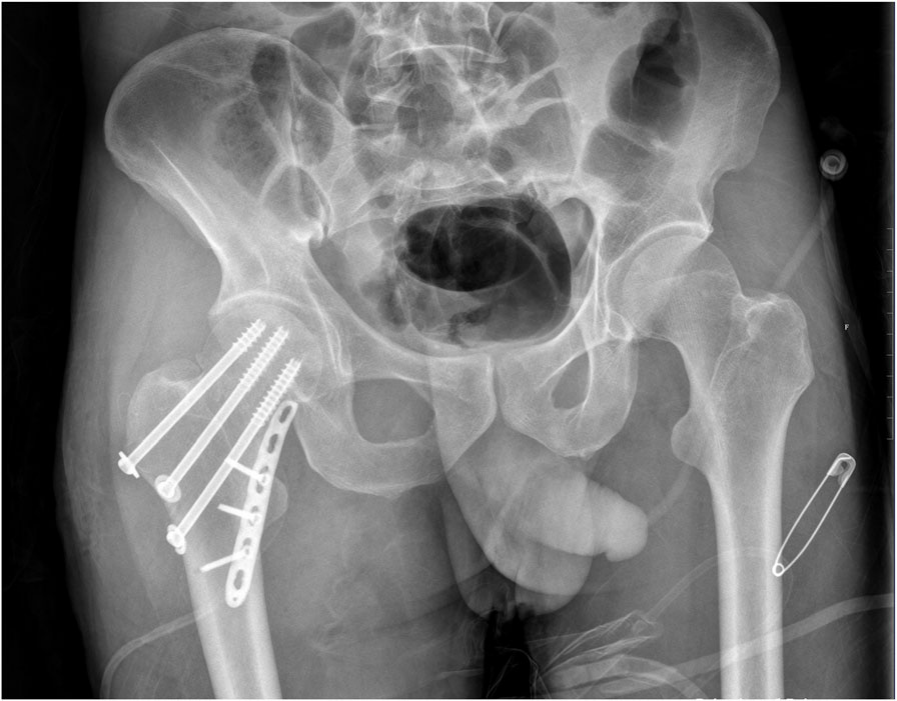
Representative X-ray image of one clinical case who was treated for vertical femoral neck fracture using three cannulated screws and a buttress plate without a proximal screw

Since the buttress plate only resists vertical shear stress and protects the cannulated screw structure from failure before fracture union, Mir and Collinge did not provide any hypotheses regarding the usage of the proximal screws [6] and proximal screws are sometimes omitted in clinical cases to reduce the dynamic compression of the cannulated screws (Fig. 5). However, other studies [7, 9] used proximal screws to improve the reduction of shear stress. Our results are in line with the latter, showing that proximal screws are important for the augmentation of vertical shear stress in vFNFs. We observed that a greater maximal stress on buttress fixation was associated with a smaller maximal stress on the cannulated screws and bone (Table 2). The maximal stress of the models that involved a proximal screw (PIIT+4HI, PIIT+6HI) was concentrated on the junction of the plate and the proximal screw, while the maximal stress of the models without a proximal screw was concentrated on the junction of the plate and distal screw (Fig. 3). This indicates that proximal screws can help to transmit force from the head to the shaft of the femur, release stress on the cannulated screws and bone, and resist shearing.

Ye et al. reported one case of implant breakage at the screw-plate junction, representing one of three cases of implant failure [9]. Our results can explain this phenomenon. There was an obvious stress concentration at the screw-plate junction in all models (Fig. 3). The MPS of the models with a proximal screw at the proximal screw-plate junction (PIIT+4HI & PIIT+6HI, 795.6 & 947.2 MPa) was even close to the yield strength of the plates (Ti-6Al-4 V-alloy, 889–921 MPa) [23]. When a proximal screw was not used, the MPS values of the buttress models (PIIT+4HI-1 & PIIT+6HI-1) were reduced and the values (294.9 & 556.2 MPa) were inferior to the yield strength. It seems that non-use of proximal screws is beneficial to prevent implant breakage. However, considering the whole structure (proximal femur, three cannulated screws, and the buttress plate), the increased MPS at the proximal screw-plate junction in the models with a proximal screw in the buttress group resulted from improved resistance of the femoral head to shear and varus displacement. This is supported by the fact that the other biomechanical parameters of the same models, such as MBS, MCS, MIFD, MBD and MID, were all reduced compared with those in the models without a proximal screw in the buttress group (Table 2). Consequently, the proximal screw on the buttress plate, acting as a load-bearing implant [24], exhibited better fixational ability in terms of stress bearing and reduction of stress and displacement on bones and cannulated screws as well as reduction of relative displacement of fracture fragments. Furthermore, maximal MPS was observed in extreme conditions where the load applied on the femoral head was three times the body weight (2100 N). This is the peak theoretical load acting on the hip joint [25], which would not occur in daily life, especially for patients who have undergone internal fixation. Therefore, the stress at the proximal screw-plate junction should be treated carefully, but should not be a complete denying factor, and proximal screws are still a good choice for the initial stability of vFNFs [7].

The sliding mechanism allows linear intraoperative and postoperative compression in the treatment of vFNFs and may facilitate fracture healing [26]. Based on this principle, the dynamic fixation, such as paralleled cannulated screws, sliding hip screws and buttress plate fixation without proximal screws, was used. However, the dynamic treatment of vFNFs is also accompanied by femoral neck shortening. As a previous study reported [27], severe fracture shortening was the most common complication identified (61%) in all failure patterns. This complication is caused by excessive resorption of bone around the fracture [28]. Severe neck shortening was classified as mechanical failure [29], as it could reduce the abductor moment and decrease the functional scores, and it may lead to local soft tissue irritation due to screws back-out [30, 31] and may even increase the risk of femoral head collapse [32]. To prevent neck shortening, one type of fixation, with the used of fully threaded screws, has been suggested by some authors [33] as a length-stable strategy. Nevertheless, it had a high risk of cutting into the acetabulum because of the absence of a sliding mechanism [29]. Another type of fixation, the use of three cannulated screws plus a buttress plate using a proximal screw, is beneficial for preventing the femoral neck shortening without the risk of cutting. Most importantly, the dominant effect of the buttress plate with a proximal screw is to resist shear force across the fracture site, which is the main biomechanical problem of vertical femoral neck fractures and may also be a key factor in femoral neck shortening. Consequently, proximal screws are a reasonable consideration for preventing femoral neck shortening in vFNFs.

When buttress plates are used in vFNFs, some drawbacks should be highlighted. First, buttress plates placed on the medial side result in a possibility of hip impingement and cases have been described in previous studies [9, 34]. Careful attention to intra-operative plate placement and avoiding the placement of buttress plates too superiorly or too anteriorly onto the femoral neck can avoid this iatrogenic impingement. However, buttress plate application should be avoided in subcapital femoral neck fractures; otherwise, the position of the plate should be as distal as possible. Transcervical and basicervical fracture patterns are more amenable patterns to be considered for the application of buttress plates, as they offer a larger footprint for hardware placement farther from the hip joint [34]. Second, for placement of the plate, an additional incision is needed, which may lead to a destruction of the blood supply. Although proper placement does not endanger blood supply to the femoral head [8], the location of the buttress plates still results in potential damage to inferior retinacular artery. Finally, the stress concentration at the screw-plate junction leads to a risk of fixation breakage [9], but if the implant is strong enough, it should not be problematic. There is no need for plate removal unless infection or nonunion necessitates total hip arthroplasty [7].

In conclusion, buttress plates improve construct mechanics, help to resist shear force and prevent varus collapse. The use of a buttress plate combined with a proximal screw shows better transmission of force and bears more stress, leading to the stress and displacement on cannulated screws and bone reduced, and finally improves overall stability of vFNFs.

There are some limitations in this study that should be acknowledged. First, the inserted parts of the screws were tied to the bone; thus, the screws could not detach from the bone under the load, which may have led to the overestimation of stiffness and the underestimation of MID. However, since all models in this study were set in the same way, between-sample comparisons would not have been affected. Second, due to the simplification of omitting the pressure capacity of the plate and the difficulty in contouring the buttress plate to the bone surface in actual practice, the fixation stability of the buttress plate was likely underestimated. The maximal improved stiffness determined in this study is smaller than that of Kunapuli SC et al. [7], in which the stiffness improved by 35% on average. However, the stiffness reported by Kunapuli SC et al. was only 959 ± 257 N/mm, which is much smaller than our study and two others [20, 21], indicating that their specific boundary condition may have overestimated the augmentation effects of the buttress plate. The purpose of our study was to analyse the function of proximal screws, therefore, these underestimations will not affect our conclusions. Third, Sawbone composite bone rather than cadaveric bone were used to represent young patients with good bone quality. Nevertheless, the stiffness of our constructs should not be considerably different than that of cadaveric bone models, as shown in the paper by Topp et al. [35]. Fourth, our models can only reflect initial stability, and the more complicated biomechanical conditions during bone healing process, such as secondary stability can hardly be addressed via current biomechanical methods. Finally, all the conclusions should be validated by further clinical studies.

## Conclusions

Based on the FEA results, buttress plates can improve construct mechanics, help to resist shear force and prevent varus collapse; under the modelling conditions, the use of a proximal screw on buttress plate may be a key technical feature in improving anti-shearing ability; additionally, this screw may be essential to reduce stress and prevent re-displacement of cannulated screws and fracture fragments.

## Data Availability

The datasets used and/or analysed during the current study available from the corresponding author on reasonable request.

## Abbreviations

vFNFs: Vertical femoral neck fractures
CS: Cannulated screw
FEA: Finite element analysis
MPS: The maximal stress of plate system
MCS: The maximal stress of cannulated screws
MBS: The maximal stress of bone
MIFD: The maximal displacement of internal fixations
MID: The maximal relative displacement of interfragments
PIIT: Inverted isosceles triangular configuration
VR: Variation rate
BV: Value for the buttress group
CV: Value for the CS group
